# Clinical Impact of “Real World Data” and Blockchain on Public Health: A Scoping Review

**DOI:** 10.3390/ijerph21010095

**Published:** 2024-01-15

**Authors:** Virginia Milone, Antonio Fusco, Angelamaria De Feo, Marco Tatullo

**Affiliations:** 1 Department of Economics, Management and Business Law, University of Bari “Aldo Moro”, P.ce Umberto I, 70121 Bari, Italy; virginia.milone@uniba.it (V.M.); angelamariadefeo69@gmail.com (A.D.F.); 2 Department of Translational Biomedicine and Neuroscience—DiBraiN, University of Bari “Aldo Moro”, P.ce G. Cesare, 70124 Bari, Italy

**Keywords:** healthcare, real-world data, big data, healthcare policies

## Abstract

The digitisation of healthcare has allowed a significant rethinking of the previous clinical protocols, improving their interoperability through substantial standardisation. These technological advances have ensured that data are comparable, as they are obtained from ‘reliable’ and certified processes; however, there are billions of data that are neither structured nor quality-controlled. They are collectively referred to as ‘Real World Data’ (RWD). Blockchain (BC) is a procedure with specific characteristics and algorithms that ensure that the stored data cannot be tampered with. Nowadays, there is an increasing need to rethink blockchain in a one-health vision, making it more than just a ‘repository’ of data and information, but rather an active player in the process of data sharing. In this landscape, several scholars have analysed the potential benefits of BC in healthcare, focusing on the sharing and safety of clinical data and its contact tracing applications. There is limited research on this matter; moreover, there is a strategic interest in managing RWD in a reliable and comparable way, despite the lack of knowledge on this topic. Our work aims to analyse systematically the most impacting literature, highlighting the main aspects of BC within the context of the new digital healthcare, and speculating on the unexpressed potential of RWD.

## 1. Introduction

Healthcare is increasingly data-focused, and as a result, a managerial bias has emerged, primarily linked to certified management and governance of digital healthcare data [1]. The concept of ‘patient-centred medicine’ is dramatically being actualized and it requires healthcare managers and clinicians to make all of these data readable and ready-to-use; nonetheless, the usefulness of the data depends on their homogeneous archiving in order to make them translatable into information usable for decision-making processes [2,3]. Nowadays, Blockchain (BC) offers a reliable and expendable option that fits well with the decentralised nature of health information. Thanks to BC, both managers and clinicians are able to obtain certified and homogeneous information from multiple independent sources, overcoming the problem of the lack of interoperability among different information systems.

In this scenario, some studies predicted how BC would be used to provide secure platforms for storing and managing a large quantity of data, not necessarily related to the major clinical demands [2,3]. The Food and Drug Administration (FDA) first used the term “Real World Data” (RWD) to refer to “...data related to patient health status and/or health care delivery, properly collected from a variety of sources” [4]. RWD*s* are thus information about care pathways and healthcare delivery, derived from a variety of institutional and non-institutional sources, such as the National Health Service (NHS) [5,6].

The European Medicines Agency (EMA) uses RWD for all of its protocols of safety evaluation, risk management, and benefit-risk assessment. RWD*s* enable the reconstruction of detailed care pathways, facilitating reliable and personalized planning of clinical management of patients [3]. The complex system of RWD*s* has the potential to significantly improve the management workflow in healthcare, but the collection and rationalization of this large amount of data implies the careful analysis of some critical issues. Such issues are primarily related to the protection of personal data; nonetheless, the current lack of interoperability among health information systems has gained attention, as it could become a technical issue that is not easy to overcome. Finally, the risk of ‘Garbage-in Garbage-out’ (GIGO), a conceptual condition based on the illogic involvement of unreliable results derived from data of low trustworthiness, is an issue mainly dependent on the proper approaches of healthcare professionals to using BC and RWD within homogeneous datasets [7].

Several researchers have analysed the potential benefits and limitations of BC in healthcare, focusing mainly on the perspective of improving individual processes. Such processes are clustered in business data processing (BDP), supply-chain management (SCM) of medical devices and pharmaceuticals, sharing of clinical evidence, and contact tracing (CT) in epidemiology [8,9,10]. In detail, the first process is BDP, which would recognize benefits from BC, as this technology is able to ensure data integrity and can provide a clear audit trail of business transactions, improving accountability and fraud prevention. Nonetheless, BC also has limitations in scalability and overall efficiency, as its performance can be affected by the volume of data and transactions, limiting scalability and efficiency [8]. The second process is SCM of medical devices, which can also benefit from BC technology. In some specific cases, it can assist in the provenance tracking and authentication of pharmaceutical products. This aid is essential to detect and prevent the circulation of counterfeit drugs, protecting patients from harm, and improving the efficiency of SCM [8,9]. Conversely, implementing blockchain for supply chain management may incur upfront costs and ongoing maintenance expenses. The third process is the sharing of clinical evidence, which has been widely improved by the use of BC in healthcare settings. Some positive aspects are related to the secure and transparent data sharing among researchers, clinicians, and regulatory bodies. This will expedite clinical research by providing a secure and efficient platform for data sharing and collaboration. Limitations of this aspect are related to the issues of obtaining patient consent and ensuring data privacy and security [9,10]. Finally, the last process involves CT in epidemiology: it has been recently considered as a strategic approach to several healthcare issues affecting large populations. BC has efficiently enhanced CT by tracking interactions and notifying individuals at risk of exposure. It can also provide real-time insights into disease transmission patterns, enabling data-driven policy decisions to optimize public health measures. As with all innovations, BC in CT may experience limitations, such as technical complexity that may require technical expertise and resources [10].

In the last decades, the increasing demand for RWD*s* from various stakeholders in the healthcare sector has led to the development of numerous technical and methodological challenges associated with the use of RWD*s* to generate Real-World Evidence (RWE). The Food and Drug Administration (FDA) has used the term “Real World Evidence” (RWE), referring to the “...clinical evidence about the usage and potential benefits or risks of a medical product derived from analysis of RWD” [4].

Thus, RWD and RWE can provide extensive information about several aspects of healthcare, and they can be used to improve health-related technologies and to ensure a better decision-making process.

However, the potential and limitations of BC technology in the management of RWD are poorly explored [11]. This scoping systematic review [12] aims to contribute to filling this gap by conducting a critical literature analysis capable of highlighting the pros and cons of BC in the field of digital healthcare through a scoping systematic review focused on the following research questions (RQ):

*RQ1: What is the state-of-the-art in the use of Blockchain in healthcare?*
*RQ2: How can RWD and BC work together to improve their clinical impact on public health?*

## 2. Relevant Sections

### 2.1. Methodology

To provide an accurate and exhaustive discussion on the literature, to address the RQ1 and RQ2, we performed a scoping review of the selected literature, following Hilary Arksey and Lisa O’Malley (2005) [12], to define the current knowledge, main limitations, potential applications, and possible future research developments [13,14] of the main topic. This approach is methodologically different from a narrative review, as we follow specific searching strategies to allow readers to replicate our analysis [15,16].

In line with the speculative nature of our hypothesis, and considering the limitations within the scientific literature sources, 3 searching strategies were performed, as follows:

- “blockchain” AND “healthcare” OR “real-world data” (searching strategy 1—SS1).
- “real-world data” AND “healthcare” (searching strategy 2—SS2).
- “real-world data” AND “innovation” (searching strategy 3—SS3).

Keywords were searched in multidisciplinary scholarly databases (DB) recognized by the scientific community: Scopus, Web of Science (WoS), and PubMed [17,18,19,20,21,22,23,24,25,26,27,28].

Only peer-reviewed journals were selected to ensure the quality of the published work [29,30]; moreover, a short timeframe of 6 years (2017–2022) was chosen, since the correlation between BC and RWD is a recent topic.

**Figure 1:**
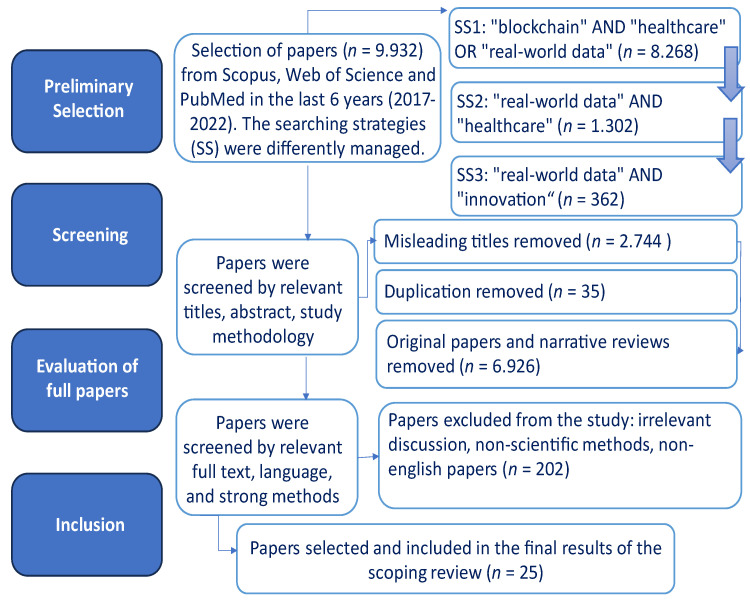
Flowchart of the methodology used in the scoping review.

The preliminary selection of papers to be included in our scoping review was based on the queries above reported, as per the SS1 (*n* = 8.268), SS2 (*n* = 1.302), and SS3 (*n* = 362). A second stage was aimed to refine our preliminary selection; here, papers were screened by relevant titles and abstracts, and we also critically evaluated the study methodology in order to include only systematic reviews in our database. The remaining articles (*n* = 227) were further considered by means of a critical reading of the full text: we carefully evaluated the methodology used and the coherence among the methods and results. The final selection identified 25 articles to be included in our scoping review (Figure 1).

Data were extracted by 2 co-authors (VM and ADF): they preliminary selected a list of articles to be examined, then they also screened the full texts of all of the included studies, independently. The data were stored in specific e-sheets (.xlm sheets), reporting the main information related to the articles, including the metadata as per PubMed format. Any final evaluation was performed in collegial brainstorming, participated in by all authors.

### 2.2. Main Advantages of Blockchain in Healthcare

Blockchain technology (BC) belongs to the broader category of distributed ledger technologies, the operation of which is primarily based on a ledger structured in blocks interconnected in a network. Each transaction executed in a block of the network of BC is validated by a process based on consensus, distributed across all nodes (i.e., devices/users connected to the network). Nevertheless, beyond its role as a secure and decentralized data repository, BC can also play an active role in streamlining and enhancing the data-sharing process in public health and healthcare. As an example, BC can facilitate the secure exchange of healthcare data between different and heterogeneous systems and stakeholders, promoting interoperability. This can significantly improve the quality of care by enabling healthcare providers to have a comprehensive view of a patient’s medical history, regardless of where they receive care.

Transactions are nothing more than the result of operations between entities within the network. Each block maintains a reference to the previous one through an encryption system, hence the concept of BC [31].

BC is not stored on a centralised server as is the case with traditional web applications but is distributed across network devices (computers) (called nodes), each of which contains a copy of the entire blockchain. As is well known, two relevant aspects that characterise BC are (i) the decentralisation of consensus and (ii) the decentralisation of ledgers. Due to the decentralisation of consensus, the existence of a fiduciary relationship between the actors involved in any type of transaction and a central authority is no longer necessary [31].

With regard to the second aspect, however, the replication and storage of multiple copies of the blockchain along the nodes of the network guarantees greater security/safety of the system and fairness between users, who will be able to have the same information at the same time, thus ensuring the traceability and immutability of the validated transactions contained in the blocks.

Blockchain technology (BC) was designed for its usefulness in ensuring privacy and reliable information, mainly in the fields of economics; nonetheless, nowadays, its utility is dramatically expanding into several other areas, including the biomedical field [11]. For example, BC can be employed to track the movement of pharmaceutical products from manufacturing to distribution to pharmacies, ensuring the authenticity and integrity of medications. Also, BC can streamline the clinical trial process by managing patient records, consent forms, and data collection, improving efficiency and compliance. It can also facilitate the identification and recruitment of eligible patients for clinical trials, accelerating the pace of drug development and the delivery of new therapies to patients.

Nowadays, there are various types of BC technologies, depending upon the information that is handled, the availability of that data, and the activities that the users are willing to do. Currently, the most well-known BCs are: (i) Public Blockchain, (ii) Private Blockchain, (iii) Consortium Blockchain, and (iv) Permissioned Blockchain. Most of them are capable of being involved in the field of medicine; intriguingly, BC has promising applications in genomics, telemedicine, tele-monitoring, neuroscience, and personalized healthcare applications.

The scientific literature has reported how the implementation of BC technologies in healthcare organisations has developed mainly in the US and the UK since 2016 [32], and BC has been mainly used for administrative data processing and supply-chain management of medical devices and pharmaceuticals to ensure compliance with regulations [33], and in the secondary use of clinical data useful for medical research [12,34].

As BC is a decentralized digital ledger used to keep medical records and information of patients in different computers, it basically aims to provide the proper place to share every kind of information within different communities. The method is simple and smart: in fact, this technology stores all data in blocks using cryptography, and only authorized users can open, read, view, and access the stored data [11].

Despite these promises seeming to be enticing, unfortunately, healthcare institutions and representatives have been, at first, reluctant to embrace the challenge of BC, even though, as some academic work has shown [35], BC technology, in its many applications, could potentially bring about countless advances in diagnosis, prevention, and medical care [36], improve the quality of the healthcare system, and facilitate the personalisation of care pathways [37].

BC can be used to collect, store, and analyse large-scale healthcare data, enabling public health officials and researchers to gain insights into population health trends, disease patterns, and the effectiveness of interventions. This can inform public health policy decisions, resource allocation, and targeted interventions to improve population health outcomes. As well as enabling cost containment in healthcare administration and improving efficiency, BC could be a valuable tool for cooperation between organisations for research and care, as it ensures privacy, reliability, and accessibility [38].

Data integrity and traceability are indeed strengths for the implementation of BC systems in healthcare [39], including disease and epidemic surveillance [40]. Patient-directed data access [41] for the sharing and management of BC-enabled Electronic Health Records (EHRs) would ensure not only protected health information, but also the ability of patients to choose and authorise the parties with whom they share data [42]. The EHRs collected as a part of routine care across clinics, hospitals, and healthcare institutions require careful and intensive pre-processing [43]. They have introduced many advantages for handling modern healthcare-related data. Nevertheless, they have created unprecedented possibilities for data-driven approaches to learn patterns, make new discoveries, enable clinical prognosis [44], enhance diagnostics, and validate and replicate clinical trial results [45].

The digitisation of healthcare through the integration of BC tools and technologies into services promises to revolutionise patient care worldwide. However, BC may not live up to its promise as it moves from testing to implementation [46]. In fact, most studies are based on the results of technical simulations and few are the result of trials in clinical settings or refer to user experiences. Many barriers to meeting the innovative challenges and opportunities that BC presents to healthcare organisations arise from the lack of specific skills among healthcare professionals, insufficient economic resources, the reluctance of patients [47] to share their health information, and the legal framework regarding the protection of individual data.

There is therefore a need for a complex, multidisciplinary, and multidimensional approach to designing innovative care models based on new services enabled by BC digital technologies that ensure a real improvement in patient care and care pathways. Some researchers have developed a model to assess the readiness of healthcare organisations to adopt BC in the context of managing electronic health record systems.

To address fundamental challenges in health data management, numerous research groups are focusing on four main areas, including (i) personalized medicine; (ii) management of privacy and data access; (iii) management of clinical information; and (iv) homogenization of clinical costs [48].

A study showed that regulatory compliance, budget availability, management support, safety, and privacy are critical to the success or failure of BC technology implementation in healthcare [49]. The Italian Superior Institute of Health (ISS), in its Strategic Plan 2021–2023, has promoted the “One Health” approach that emphasises the inextricable link between human health and the health of the entire ecosystem.

Interaction between professional groups and the integration of expertise would be enhanced by extending and improving the efficiency of existing networks. BC technologies could support the evolution of healthcare organisations towards the Connected Care organisational model. By overcoming critical security, interoperability, and privacy issues, BC would unlock its potential and enable healthcare organisations to meet the One Health challenge.

**Figure 2:**
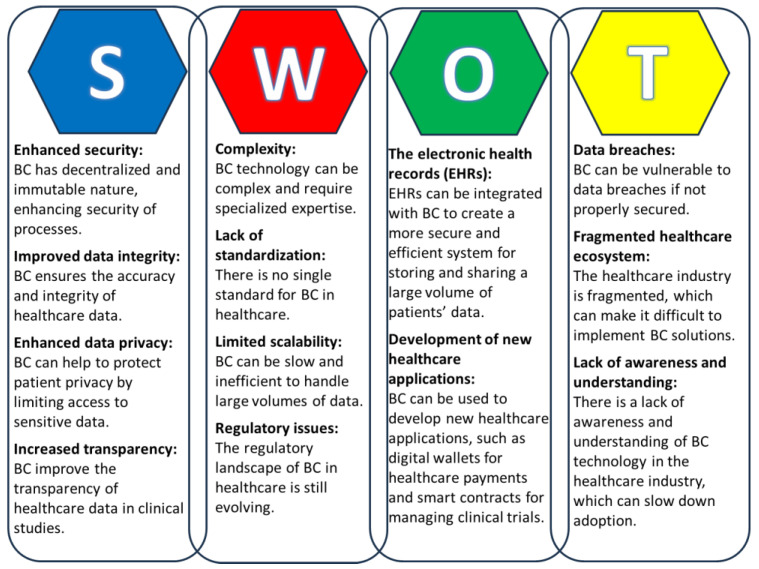
SWOT analysis of the use of Blockchain technology in healthcare.

Overall, BC technology has several strengths and holds the potential to revolutionize the healthcare industry by providing a more secure, efficient, and transparent way to store, share, and manage patient data. However, there are a number of challenges that need to be addressed before blockchain can be widely adopted in healthcare. These challenges include complexity, lack of standardization, scalability, and regulatory uncertainty (Figure 2).

### 2.3. RWD and BC: Should We Use Them in Public Health and Healthcare Management?

The academic literature on the implementation of Health Information Technology (HIT) systems in healthcare organisations suggests the Technology Acceptance Model (TAM) as a model for the evaluation and acceptance of technology by managers, healthcare professionals, and patients [50]. This model is based on a positivist view that considers the relationship between technology and health mainly in terms of causal and linear benefits [51]. The TAM can be effectively employed to evaluate and assess the acceptance of HIT systems among healthcare managers, healthcare professionals, and patients. TAM suggests that perceived ease of use (PEOU) and perceived usefulness (PU) are crucial factors influencing user acceptance and utilization of HIT systems. Healthcare organizations can enhance PEOU and PU by designing systems for intuitiveness, emphasizing system benefits, providing comprehensive training and support, and involving users in the decision-making process. By adopting these TAM principles, healthcare organizations can foster higher HIT adoption rates and reap the benefits of improved healthcare delivery and outcomes [50,51].

From this perspective, the use of technology solutions is useful not only to improve business processes, care pathways, and clinical research, but also to solve complex social problems [5,6]. In this view, BC is able to innovate and improve business processes by providing managers, clinicians, and patients with a single, up-to-date, and immutable repository of data on care pathways and healthcare delivery.

Blockchain-based RWD systems hold great potential to rebuild healthcare; up to now, their implementation has required a multi-pronged approach that encompasses organizational changes and effective change management strategies. Healthcare organizations must integrate RWD seamlessly with existing healthcare systems for efficient data flow and interoperability. To safeguard patient privacy and comply with regulatory requirements, organizations must implement robust data privacy and security measures. Additionally, they must develop secure mechanisms for sharing RWD among authorized parties, such as healthcare providers, researchers, and regulatory agencies [7,8,9,10,11].

The successful implementation of blockchain-based RWD systems also hinges on effective change management strategies. Executive buy-in is crucial to drive change and foster buy-in across the organization [47]. Clear communication of the benefits of blockchain-based RWD systems, addressing concerns and providing transparency, is essential. Pilot projects have provided a controlled environment to test the blockchain system, gathering valuable feedback before full-scale deployment. Incentive programs can further encourage adoption and participation in the blockchain-based RWD system [51].

Real-world data, with its rich insights into patient outcomes and treatment effectiveness, could be used in several business cases. Blockchain-based RWD systems are used in precision medicine, to identify patterns and risk factors for specific diseases, enabling personalized treatment approaches; moreover, drug discovery and development can be accelerated by analysing RWD to identify potential drug candidates and monitor their efficacy and safety in real-world settings, reducing the time and cost of drug development.

By embracing effective change management strategies and addressing organizational challenges, healthcare organizations can unlock the transformative potential of blockchain-based RWD systems, revolutionizing patient care, advancing medical research, and promoting public health initiatives (Table 1).

BC is based on a decentralised structure, and it ensures that data are protected from any point of view, making BC much more secure than traditional centralised storage systems. Due to the decentralised and tamper-proof nature of the records used by BC, this allows healthcare professionals to easily make changes to such data in real time; nonetheless, this also ensures that all of the parties involved within the BC process have access to the most relevant and up-to-date information [41,42,43,47]. This may not mislead readers; in fact, BC merges the widest data sharing with the strictest maintenance of the privacy of patients. In fact, the data are encrypted and can only be accessed by authorised parties, thus ensuring the confidentiality of sensitive information [11,12,13,48]. Additionally, BC makes it easier for medical records to be shared securely and efficiently between healthcare professionals, and this also helps to promote a more collaborative and coordinated approach to patient care [38,41].

The legal framework for the protection of privacy is constantly evolving to ensure that data processing complies with current best practices, and it is strategic to use technologies that guarantee data security and accessibility [49]. The establishment of a risk-benefit profile is a central part of the regulatory process for assessing the safety of medicines. Data management has become a critical issue in the biomedical field. These data are typically stored in centralised databases, which creates vulnerabilities and is susceptible to cyber-attacks. In the last decade, many healthcare organisations have been victims of preventable cybersecurity attacks [51].

Additionally, data heterogeneity is another challenge in RWD analysis. The huge size and highly heterogeneous nature of healthcare data make it relatively less informative using traditional technologies. In this scenario, BC can help guard against the risk of data theft or tampering, thanks to its immutability feature based on cryptographic principles. Health data stored on BC is also safe from damage because it is stored in multiple locations [51].

The concept of sustainability is a relatively new area of study, with most of the research coming from the healthcare sector. Healthcare sustainability and technological advancement initiatives are not yet widespread, but there is a growing belief within healthcare organisations that there is value in achieving long-term sustainability goals, as well as an awareness that digital transformation enables sustainable models for healthcare delivery. In this context, it is important to recognise that sustainability is an issue that is inherently linked to health and well-being. Numerous factors have been suggested to promote sustainability, such as the effectiveness of the intervention, its characteristics, and its costs [38]. The economic and qualitative state of the NHS calls for consideration of the possibility of continuing to provide such a wide range of health and social care services without being able to ensure that the quality of the services provided does not suffer [6]. Therefore, it is important to reflect on the sustainability of the national health system, which has already shown signs of inefficiency over the last decade [48,49,50]. Furthermore, one of the most important aspects of the sustainability of the healthcare system is the reduction of waste and inefficiency, which account for a large proportion of the resources allocated to the system and which undoubtedly worsen the performance of the system as a whole [6].

The decentralised nature of registries allows data from multiple heterogeneous information sources to be entered and shared in the system and to relate to ‘real-world’ contexts, even decontextualised from the clinical focus, known as real-world data (RWD). The main sources of RWD are information flows from the National Health Service (e.g., flows related to territorial medicines, SDOs, outpatient services, and telemedicine services), other institutional (e.g., EHR, AIFA registers, databases managed by INAIL and INPS, and ISTAT surveys) and non-institutional sources (e.g., data obtained directly from patients, scientific societies, and professional networks) and non-randomised clinical trials [54].

RWD*s*, suitably processed and systematised, can thus be shared for clinical and management purposes, supporting complex decision-making processes within healthcare organisations and the system as a whole. RWD streams derived from multiple sources of information can be used to reconstruct a patient’s treatment pathway and, consequently, the organisation’s production processes, facilitating the control of the appropriateness of services provided, health planning activities, health technology assessment, and clinical research.

In this scenario, several studies have already suggested the application of blockchain for better management and sharing of patient data from NHS information flows, particularly from EHR/EMRs (electronic health and medical records) [12,28,52,54,55,56,57]. Managing data from EHRs with the blockchain system could reduce clinical bias and provide a powerful clinical risk management tool for practitioners, while reducing the ‘Garbage-in Garbage-out’ (GIGO) phenomenon. Garbage-in Garbage-out, literally the production of unreliable results from low-reliability data, occurs most commonly when RWD is used in large population studies [58]. This phenomenon is mitigated by the BC system, which is able to guarantee the provenance of the data, its integrity, and its immutability.

## 3. Discussion

Generating reliable evidence on the designs and effectiveness of disease prevention, diagnosis, and treatment in real-world settings is a priority for researchers, healthcare providers, and regulators. In this scenario, Real-World Data (RWD), i.e., data on patient health and healthcare delivery from routinely collected sources, can be an important component for addressing a number of significant research questions in healthcare.

Nowadays, various biomedical and healthcare tools generate a large amount of data. Therefore, it is mandatory for us to know the enormous potential of such composite datasets in the light of data mining and data fusion processes. However, the management of RWD*s* in healthcare undoubtedly raises some relevant critical issues, such as (i) the management of sensitive data allowing the identification of individual patients and their health status; (ii) the current lack of interoperability of information systems from which data are extracted; (iii) the complexity of data collection and attribution processes and the confusion of their taxonomy, which is often far from unambiguous, with the risk of a “Garbage-in Garbage-out” situation [7]. In addition, there is a lack of consensus on the appropriate Data Governance (DG) practices for RWD/RWE. Data sharing is also a major concern, particularly in light of evolving data protection regulations.

As is well known, the decentralised and transparent nature of this technology raises, in certain application contexts, issues related to privacy and network security (in many ways still unresolved and debated), in particular with regard to the exchange of sensitive data in public blockchains. Considering, for example, the regulation of data protection governed by the General Data Protection Regulation (GDPR) at the European level, a not insignificant aspect is a way to identify data controllers and data processors while ensuring the comprehensive protection provided by the same regulation for the parties involved in the network [53,59,60]. Therefore, while decentralisation and immutability, which are typical characteristics of BC, on the one hand, allow for effective transparency and security in the acquisition and exchange of RWD; on the other hand, they may constitute a point of conflict with existing legislation.

The literature on this subject does not show any concrete approach to the problem of privacy protection; the potential solutions hypothesised here converge on two aspects that characterise BC and that could reduce the problem of data protection: the decentralisation of consent and the cryptographic system [2,46,61,62].

The immutability features of distributed registries and the security of the information exchanged within the BC system could solve the problem associated with the current lack of interoperability of health information systems, thus facilitating clinical decisions [58].

Patients will only benefit from digital tools if health systems support digital inclusion policies. As healthcare increasingly moves towards a ‘digital first’ approach, digital inclusion is becoming intertwined with the broader concept of healthcare equity.

Furthermore, in a highly regulated sector such as healthcare, the use of a single decentralised platform capable of ‘certifying’ the provenance of the data collected would drastically reduce the risk of a GIGO situation.

The standardized use of BC technology provides a transparent and secure method to process big data with overall safety and at a lower cost. Furthermore, the use of RWD can make it possible to estimate the costs of therapies and the overall costs of disease management, and this choice makes us able to decide how to optimally allocate resources for the screening, prevention, and treatment of specific diseases [63].

It is now clear that digital healthcare tools bring substantial benefits to the entire healthcare system, and that the direction taken by major healthcare facilities is to ensure maximum reliability, punctuality, and security, avoiding the waste of time and precious information. In this scenario, the “*connected care*” concept aims to connect the patient with healthcare personnel involved in the entire care pathway. Obviously, BC technology is the key tool that enables this integration, allowing data to be shared and not wasted. Especially in RWD management, it is of pivotal importance that there are data protection laws in place that adequately protect the privacy of patients. Due to the confidential nature of clinical data, ensuring data security is an urgent issue in the development of a digital healthcare system [58].

Blockchain also means “automation”, and where there is automation, there are savings. However, it is possible to reduce the administrative complexity of healthcare, and thanks to BC, processes can be automated easily and efficiently while remaining secure. RWDs have the potential to reduce inefficiencies and fill gaps in the management of information generated by all stakeholders in the patient journey: healthcare companies, pharmaceutical companies, government agencies, and patients [42,43,44]. This sharing of information and data in turn enables all parties to gain new insights, support value-based care, and deliver better health outcomes [11,64].

As reported by the FDA, the use of RWD and RWE has several positive spin-offs for those managing the patient care pathway, for pharmaceutical companies, and, last but not least, for the patients themselves [4,54]. While helping to monitor safety, they also enable more informed evaluation of guidelines and appropriate clinical decisions, allowing for new treatment pathways and clinical trials that are increasingly tailored to the specific health profile of the patient. Moreover, RWD management represents a valuable tool for the pharmaceutical industry in demonstrating the value of manufactured medicines throughout their life cycle, from clinical trials to market maturity [54].

Regardless of the reliability and cross-checking of these groups, BC is a viable approach to securely sharing data between different groups of people. Nevertheless, the combination of BC and machine learning systems will be able to generate data that can be used to create predictive models that are useful for risk management [53].

## 4. Conclusions

The use of real-world data (RWD) has the potential to fill current gaps in the healthcare system’s knowledge and management of certain diseases and also to identify gaps between evidence and practice, ultimately leading to improved care and patient outcomes.

To conclude this study, a summary of the main potentials and limitations of BC technology in the use of RWD from a managerial perspective is presented below, in Table 2.

Briefly, the main potentials of BC technology in the use of Real-World Data (RWD) are represented by the ability of these systems to speak the same language by means of the interoperability of health information systems: this dialogue improves multidisciplinary management, thus reducing the GIGO bias, and increases the clinical outcomes useful in clinical settings. Nonetheless, our research has identified some important limitations. In this regard, it should be noted that BC technology is a new way to do healthcare. This could mean that there is a large number of professionals not ready to use it properly. Importantly, legal aspects related to these technologies have not been properly addressed and defined, and there is a lack of studies or projects demonstrating a concrete benefit from the use of RWD and BC in healthcare. In the authors’ opinion, the approach reported here cannot be considered the most predictable one, so its use and development in a fully operative environment like healthcare could significantly alter the research findings in different settings. Finally, the relationship between BC and RWD is still an under-researched topic, as evidenced by the lack of research on the subject, despite the authors’ systematic use of the main scientific research databases.

## 5. Future Directions

Achieving a more efficient health system is a necessary step, and many of the solutions adopted need to become systemic in order to bring about a transformation towards more advanced and sustainable healthcare.

As the healthcare industry embraces the transformative potential of blockchain technology, increasingly smart interactions among healthcare professionals and stakeholders are required for data management, patient care, and clinical approach standardization. On the other hand, while BC and RWD are safe for several data mining and fusion procedures, they cannot ensure the immutability of smart contracts once deployed on the blockchain, as vulnerabilities may affect their correctness, particularly when dealing with sensitive patient data [65].

In response to these challenges, formal methods—a suite of rigorous mathematical techniques—have emerged as a promising solution [66]. These methods provide a systematic approach to verifying smart contract code and mitigating the risk of costly errors and security breaches. By employing formal methods, developers can ensure the confidentiality, integrity, and availability of sensitive patient data, safeguard the immutability of medical records, and prevent potential fraud or misuse of healthcare resources.

In the context of healthcare smart contracts, formal methods can be employed to ensure adherence to data privacy regulations and ethical considerations and to formally verify contract correctness, ensuring they meet the specified properties and adhere to healthcare standards [65,66].

Considering the main potentials, but also the overall limitations, possible future directions for the implementation of BC in RWDs management could be as follows: (i) open standards (anonymous data sharing); (ii) national policies; (iii) patient control, privileges, and access; (iv) artificial intelligence (AI) integration; and (v) automation and microservices.

Therefore, this work serves as a basis for a larger and more structured study to better generalise the findings. Future research could investigate, through the analysis of business cases, the organisational changes required to implement a blockchain system using RWD*s* and the associated change management strategies to be implemented.

## Figures and Tables

**Table 1:** Comparative overview of the main studies focused on Blockchain (BC) technology in healthcare and public health.

First Author (Year)	Aim/Scope of the Article	BC Application	Ref.
Motsi-Omoijiade, I. (2021)	Blockchain for healthcare applications and use cases.	Supply Chain Management	[32]
Durneva, P. (2020)	The current state of research, challenges, and future research directions of blockchain technology in patient care.	Personalized Medicine	[35]
Orcutt, M. (2017)	Why the CDC wants in on blockchain.	Data Sharing and Access	[36]
Almalki, T. (2021)	Healthcare Security based on Blockchain.	Patient Identity Management	[37]
Stamatellis, C. (2020)	A privacy-preserving healthcare framework using hyperledger fabric.	Clinical Trials	[38]
Wachter, R. (2015)	The digital doctor. Hope, hype and at the dawn of medicine’s computer age.	Data Governance	[39]
Reegu, F.A. (2021)	A systematic review of the benefits and threats of blockchain technology in healthcare.	Payer-Provider Collaboration	[40]
Abbas, K. (2020)	A blockchain and machine learning-based drug supply chain management and recommendation system for smart pharmaceutical industry.	Payments and Reimbursement	[41]
Saeed, H. (2022)	Blockchain technology in healthcare and regulatory aspects.	Regulatory Compliance	[42]
Khatoon, A. (2020)	A BC-based smart contract system for healthcare management.	Remote Patient Monitoring	[52]
Fusco, A. (2020)	Blockchain in healthcare: Insights on COVID-19.	Contact Tracing in pandemics	[53]

**Table 2:** The main potentials and limits of Blockchain (BC) technology in the use of Real-World Data (RWD) in a managerial perspective related to healthcare and public health.

Main Potentials	Main Limits
Interoperability among healthcare systems	Resistance to change by health professionals
Reliable support for decision-making processes	Lack of specific skills in healthcare workers
Improvement of care outcomes	Limitations may arise from legal aspects
Reduction of Garbage-in Garbage-out (GiGo)	Only a few studies reported benefits from the use of BC in public health
Increased transparency of care outcomes	BC is not fully mature and has not been investigated in healthcare settings

## Data Availability

No new data were created or analyzed in this study. Data sharing is not applicable to this article.

## References

[1] Evangelatos N., Özdemir V., Brand A. (2020). Blockchain for digital health: Prospects and challenges. OMICS.

[2] Vazirani A.A., O’Donoghue O., Brindley D., Meinert E. (2019). Implementing blockchains for efficient health care: Systematic review. J. Med. Internet Res..

[3] Rudrapatna V.A., Butte A.J. (2020). Opportunities and challenges in using real-world data for health care. J. Clin. Investig..

[4] Schurman B. (2019). The Framework for FDA’s Real-World Evidence Program. https://www.fda.gov/media/120060/download.

[5] Dreyer N.A. (2018). Advancing a framework for regulatory use of real-world evidence: When real is reliable. Ther. Innov. Regul. Sci..

[6] Perniconi B., Coletti D., Aulino P., Costa A., Aprile P., Santacroce L., Chiaravalloti E., Coquelin L., Chevallier N., Teodori L. (2014). Muscle acellular scaffold as a biomaterial: Effects on C2C12 cell differentiation and interaction with the murine host environment. Front Physiol..

[7] Kilkenny M., Robinson K.R. (2018). Data quality: “Garbage in-garbage out”. Health Inf. Manag..

[8] Aloini D., Latronico L., Pellegrini L. (2022). The impact of digital technologies on business models. Insights from the space industry. Measur. Bus. Excell..

[9] Chenthara S., Ahmed K., Wang H., Whittaker F., Chen Z. (2020). Healthchain: A novel framework on privacy preservation of electronic health records using blockchain technology. PLoS ONE.

[10] Kraus S., Schiavone F., Pluzhnikova A., Invernizzi A.C. (2021). Digital transformation in healthcare: Analyzing the current state-of-research. J. Bus. Res..

[11] Kuo T.-T., Kim H.-E., Ohno-Machado L. (2017). Blockchain distributed ledger technologies for biomedical and health care applications. J. Am. Med. Inform. Assoc..

[12] Arksey H., O’Malley L. (2005). Scoping studies: Towards a methodological framework. Int. J. Social. Res. Methodol..

[13] Massaro M., Dumay J., Guthrie J. (2016). On the shoulders of giants: Undertaking a structured literature review in accounting. Account. Audit. Account. J..

[14] Parris D.L., Peachey J.W. (2013). A Systematic Literature Review of Servant Leadership Theory in Organizational Contexts. J. Bus. Ethics.

[15] Parada M., Nordqvist M., Gimeno A. (2010). Instituzionalizing the family business: The role of professional associations in fostering a change of values. Fam. Bus. Rev..

[16] Cronin P., Ryan F., Coughlan M. (2008). Undertaking a literature review: A step-by-step approach. Br. J. Nurs..

[17] Brzezinski M. (2015). Power laws in citation distributions: Evidence from Scopus. Scientometrics.

[18] Zhu J., Liu W. (2020). A Tale of Two Databases: The Use of Web of Science and Scopus in Academic Papers. Scientometrics.

[19] Li K., Rollins J., Yan E. (2018). Web of Science Use in Published Research and Review Papers 1997–2017: A Selective, Dynamic, Cross-Domain, Content-Based Analysis. Scientometrics.

[20] Van Eck N.J., Waltman L. Accuracy of Citation Data in Web of Science and Scopus. Proceedings of the 16th International Conference on Scientometrics and Informetrics (ISSI 2017).

[21] Visser M., Jan Van Eck N., Waltman L. (2020). Large-Scale Comparison of Bibliographic Data Sources: Scopus, Web of Science, Dimensions, Crossref, and Microsoft Academic. arXiv.

[22] Vera-Baceta M.A., Thelwall M., Kousha K. (2019). Web of Science and Scopus Language Coverage. Scientometrics.

[23] Huang C.-K., Neylon C., Brookes-Kenworthy C., Hosking R., Montgomery L., Wilson K., Ozaygen A. (2020). Comparison of Bibliographic Data Sources: Implications for the Robustness of University Rankings. Quant. Sci. Stud..

[24] Martín-Martín A., Orduna-Malea E., Thelwall M., López-Cózar E.D. (2018). Google Scholar, Web of Science, and Scopus: A Systematic Comparison of Citations in 252 Subject Categories. J. Informetr..

[25] Wouters P., Thelwall M., Kousha K., Waltman L., de Rijcke S., Rushforth A., Franssen T. (2015). The Metric Tide: Literature Review (Supplementary Report I to the Independent Review of the Role of Metrics in Research Assessment and Management).

[26] Mongeon P., Paul-Hus A. (2016). The Journal Coverage of Web of Science and Scopus: A Comparative Analysis. Scientometrics.

[27] Pranckute R. (2021). Web of Science (WoS) and Scopus: The Titans of Bibliographic Information in Today’s Academic World. Publications.

[28] Kim W., Yeganova L., Comeau D.C., John Wilbur W., Lu Z. (2022). Towards a unified search: Improving PubMed retrieval with full text. J. Biomed. Inform..

[29] Miller C.C. (2006). Peer review in the organizational and management sciences: Prevalence and effects of reviewer hostility, bias, and dissensus. Acad. Manag. J..

[30] Easterby-Smith M., Thorpe R., Jackson P. (2019). Management Research.

[31] Hughes A., Park A., Kietzmann J., Archer-Brown C. (2019). Beyond Bitcoin: What blockchain and distributed ledger technologies mean for firms. Bus. Horiz..

[32] Motsi-Omoijiade I., Kharlamov A. (2021). Blockchain for healthcare applications and use cases. Blockchain Public. Law..

[33] Curran K. (2019). Transforming Industry and Society: Blockchain beyond the Coin, Information Age. https://www.information-age.com/transforming-industry-society-blockchain-beyond-coin-16407/.

[34] Yeung K. (2021). The health care sector’s experience of blockchain: A cross-disciplinary investigation of its real transformative potential. J. Med. Internet Res..

[35] Durneva P., Cousins K., Chen M. (2020). The current state of research, challenges, and future research directions of blockchain technology in patient care: Systematic review. J. Med. Internet Res..

[36] Orcutt M. Why the CDC wants in on blockchain. *MIT Technol. Rev.*
**2017**. https://www.technologyreview.com/2017/10/02/148864/why-the-cdc-wants-in-on-blockchain/.

[37] Almalki T., Alzahrani S., Alhakami W. (2021). Healthcare Security based on Blockchain. Int. J. Comput. Sci. Net. Sec..

[38] Stamatellis C., Papadopoulos P., Pitropakis N., Katsikas S., Buchanan W.J. (2020). A privacy-preserving healthcare framework using hyperledger fabric. Sensors.

[39] Wachter R. (2015). The digital doctor. Hope, Hype and at the Dawn of Medicines Computer Age. Malays. Orthop. J..

[40] Reegu F.A. (2021). A systematic review of benefits and threats of blockchain technology in Healthcare. IJETMS.

[41] Abbas K., Afaq M., Ahmed Khan T., Song W.C. (2020). A blockchain and machine learning-based drug supply chain management and recommendation system for smart pharmaceutical industry. Electronics.

[42] Saeed H., Malik H., Bashir U., Ahmad A., Riaz S., Ilyas M., Bukhari W.A., Khan M.I.A. (2022). Blockchain technology in healthcare: A systematic review. PLoS ONE.

[43] Sun W., Cai Z., Li Y., Liu F., Fang S., Wang G. (2018). Data processing and text miming technologies on electronic medical records: A review. J. Health Eng..

[44] Mahmoudi E., Kamdar N., Kim N., Gonzales G., Singh K., Waljee A.K. (2020). Use of electronic medical records in development and validation risk prediction models of hospital and readmission: Systematic review. BMJ.

[45] Bartlett V.L., Dhruva S.S., Shah N.D., Ryan P., Ross J.S. (2019). Feasibility of using real-world data to replicate clinical trial evidence. JAMA Netw. Open.

[46] El-Gazzar R., Stendal K. (2020). Blockchain in health care: Hope or hype?. J. Med. Internet Res..

[47] Esmaeilzadeh P., Mirzaei T. (2019). The potential of blockchain technology for health information exchange: Experimental study from patients’ perspectives. J. Med. Internet Res..

[48] Engelhardt M.A. (2017). Hitching Healthcare to the Chain: An Introduction to Blockchain Technology in the Healthcare Sector. Tecnhol. Innov. Manag. Rev..

[49] Alzahrani S., Daim T., Choo K.K.R. (2022). Assessment of the blockchain technology adoption for the management of the electronic health record systems. IEEE Trans. Eng. Manag..

[50] Holden R.J., Karsh B.T. (2010). The technology acceptance model: Its past and its future in health care. J. Biomed. Inform..

[51] Greenhalgh T., Swinglehurst D., Stones R. (2014). Aims, approach and research questions. In Rethinking resistance to ‘big IT’: A sociological study of why and when healthcare staff do not use nationally mandated information and communication technologies. NIHR J. Libr..

[52] Khatoon A. (2020). A blockchain-based smart contract system for healthcare management. Electronics.

[53] Fusco A., Dicuonzo G., Dell’Atti V., Tatullo M. (2020). Blockchain in healthcare: Insights on COVID-19. Int. J. Environ. Res. Public Health.

[54] Sherman R.E., Anderson S.A., Dal Pan G.J., Gray G.W., Gross T., Hunter N.L., Califf R.M. (2016). Real-world evidence—What is it and what can it tell us?. N. Engl. J. Med..

[55] Rabah K. (2017). Challenges & opportunities for blockchain powered healthcare systems: A review. J. Med. Health Sci..

[56] Zhong J., Zhang J., Fang H., Liu L., Xie J., Wu E. (2022). Advancing the development of real-world data for healthcare research in China: Challenges and opportunities. BMJ Open.

[57] Kothari P., Nuce M., Vasiliu-Feltes I., Hurley D., Fox M., Sneha S., Iyengar R. (2021). Blockchain predictions for health care in 2021. Blockchain Healthc. Today.

[58] Angeles R. (2019). Blockchain-based healthcare: Three successful proof-of-Concept pilots worth considering. J. Int. Technol. Inf. Manag..

[59] Drescher D. (2017). Blockchain Basics: A Non-Technical Introduction in 25 Steps.

[60] Venier F. (2018). La Blockchain oltre il Bitcoin. Cos’è e cosa può fare per le aziende. Sist. Impresa.

[61] Fatima N., Agarwal P., Shahab S.S. (2022). Security and Privacy Issues of Blockchain Technology in Health care—A Review. ICT Analysis and Applications.

[62] Lee T.F., Chang I., Kung S.T. (2021). Blockchain-Based Healthcare Information Preservation Using Extended Chaotic Maps for Hipaa privacy/security Regulations. Appl. Sci..

[63] Ramzan S., Aqdus A., Ravi V., Koundal D., Amin R., Al Ghamdi M.A. (2022). Healthcare Applications Using Blockchain Technology: Motivations and Challenges. IEEE Trans. Eng. Manag..

[64] Goldberg R.M., Wei L., Fernandez S. (2017). The evolution of clinical trials in oncology: Defining who benefits from new drugs using innovative study designs. Oncologist.

[65] Krichen M., Lahami M., Al–Haija Q.A. Formal Methods for the Verification of Smart Contracts: A Review. Proceedings of the 15th International Conference on Security of Information and Networks (SIN).

[66] Abdellatif T., Brousmiche K. Formal Verification of Smart Contracts Based on Users and Blockchain Behaviors Models. Proceedings of the 9th IFIP International Conference on New Technologies, Mobility and Security (NTMS).

